# The Initial COVID-19 Reliable Interactive DNA Methylation Markers and Biological Implications

**DOI:** 10.3390/biology13040245

**Published:** 2024-04-07

**Authors:** Zhengjun Zhang

**Affiliations:** School of Computer, Data and Information Sciences, University of Wisconsin, Madison, WI 53706, USA; zjz@stat.wisc.edu

**Keywords:** causative effects, SARS-CoV-1, MERS-CoV, SSPE, influenza, diabetes

## Abstract

**Simple Summary:**

A fundamental scientific question—where did SARS-CoV-2 come from?—has eluded the scientific community since it was first identified in December 2019. SARS-CoV-2/COVID-19 is still infecting humans. Like many other viruses, SARS-CoV-2 has been regarded as an RNA virus. However, the pathological knowledge of the cause of COVID-19 and the intrinsic drivers of virus replications are unknown. Finding an answer can help in understanding the virus and preventing the next pandemic. Many COVID-19 research results at the genomic level have been published in the literature. These published results have explored the pathological causes of COVID-19 infection from various aspects. Due to the limitations of research methodology, some published results can hardly be cross-validated from cohort to cohort. As a result, there is still an urgent need to study the root causes further. We aim to find an answer to the fundamental scientific question. We use a new AI algorithm to identify critical genes at the DNA methylation level. Our results are computational with biological implications. They are interpretable, accurate, and cross-validated. They can be reproduced from Excel sheets using the derived formula. Our findings demand rigorous and much deeper study.

**Abstract:**

Earlier research has established the existence of reliable interactive genomic biomarkers. However, reliable DNA methylation biomarkers, not to mention interactivity, have yet to be identified at the epigenetic level. This study, drawing from 865,859 methylation sites, discovered two miniature sets of Infinium MethylationEPIC sites, each having eight CpG sites (genes) to interact with each other and disease subtypes. They led to the nearly perfect (96.87–100% accuracy) prediction of COVID-19 patients from patients with other diseases or healthy controls. These CpG sites can jointly explain some post-COVID-19-related conditions. These CpG sites and the optimally performing genomic biomarkers reported in the literature become potential druggable targets. Among these CpG sites, cg16785077 (gene *MX1*), cg25932713 (gene *PARP9*), and cg22930808 (gene *PARP9*) at DNA methylation levels indicate that the initial SARS-CoV-2 virus may be better treated as a transcribed viral DNA into RNA virus, i.e., not as an RNA virus that has concerned scientists in the field. Such a discovery can significantly change the scientific thinking and knowledge of viruses.

## 1. Introduction

The pathological knowledge of the cause of COVID-19 and the intrinsic drivers of virus replications are unknown, at least at the genomic and DNA methylation levels. However, many research papers have targeted these urgent needs [1,2,3,4,5,6,7]. In the literature, on the one hand, the earlier work [8,9,10], with the highest possible accuracy, first discovered that the genomic representation geometry spaces between SARS-CoV-2 (NP/OP PCR swabs) and COVID-19 (blood samples) are significantly different at the genomic level. Using the set of optimum interactive genomic biomarkers, the work studying vaccine effectiveness found adverse effects in gene expressions from taking the BNT162b2 vaccine within COVID-19-convalescent octogenarians [11]. In an unpublished circulated note within a medical research group, adverse effects were also found in gene expressions from taking inactivated vaccines, using GSE189263 data [12]. On the other hand, the role of methylation in gene expression has drawn attention in disease studies [13]. Significant empirical evidence exists such that errors in methylation could be responsible for diseases, which has motivated studying COVID-19 at DNA methylation levels. However, COVID-19 DNA methylation studies are relatively sparse compared to gene transcriptomic data analysis. The following are some examples. Balnis et al. conducted genome-wide DNA methylation analysis of COVID-19 severity and COVID-19 free from respiratory symptoms using whole blood samples [14]. Davalos et al. studied the DNA methylation status of whole blood samples from pediatric COVID-19 cases and healthy control cases [3]. Konigsberg et al. studied methylation predicting SARS-CoV-2 infection and the clinical outcomes [5]. Morselli et al. conducted targeted DNA methylation profiling in a cohort of pneumonia patients and unaffected individuals from peripheral blood [6]. This paper intends to identify COVID-19’s optimum interactive DNA methylation biomarkers.

A critical characteristic of reliable DNA methylation biomarkers is that they hold intrinsic and robust properties for different trials and cohorts. They lead to an overall accuracy of 95% or higher among all cohorts available for testing, with some cohorts having 100% accuracy. They are independent of extrinsic characteristics. Indeed, finding such reliable biomarkers is rather challenging. Many published gene biomarkers derived from a single trial (cohort) cannot be applied to other trials, or sometimes only with low efficiency. Using breast cancer diagnosis as an example, the known eight famous genes—*BRCA1*, *BRCA2*, *PALB2*, *BARD1*, *RAD51C*, *RAD51D*, and *ATM*—were shown to perform with low efficiency; see the published paper [15] and the references therein. These drawbacks raise outstanding concerns about many published gene biomarkers, i.e., they should not be used as biomarkers as they can mislead in the wrong direction and mask the truth. One possible reason for the claimed biomarkers’ failure to be valid may be the limitations of the analysis method and tools. A fundamental flaw is that the published gene biomarkers did not exhibit interaction with each other and with the disease subtypes which are defined in this paper, and as a result, their usefulness can be somewhat limited. In this paper, we target finding reliable DNA methylation biomarkers.

A fundamental question—where did SARS-CoV-2 come from?—has eluded the scientific community since it was first identified in December 2019. Like many other viruses, SARS-CoV-2 has been regarded as an RNA virus [16]. A comparison study of the viral phylogenetic trees of known pandemic coronaviruses (SARS-CoV, MERS-CoV, and SARS-CoV-2), as well as pandemic influenza A strains (H1N1, H3N2, and H5N1), revealed that SARS-CoV-2 is most closely related to SARS-CoV and MERS-CoV [17]. In this paper, we found that DNA methylations’ marker genes *MX1* and *PARP9* identified for SARS-CoV-2 have been rooted in SARS-CoV and MERS-CoV [18,19,20], which indicates that the initial SARS-CoV-2 may be better treated as a transcribed viral DNA into RNA virus due to the likely long incubation feature of *MX1* associated diseases, i.e., not as a positive-strand RNA virus as other scientists have thought. Such a discovery can significantly change the scientific thinking and knowledge of viruses.

In this paper, we applied a proven, powerful analytical approach to identify nearly perfect interactive DNA methylation biomarkers for COVID-19 [8,9,10,11,15,21,22,23]. The significant contributions of this paper are six-fold: (1) A discovery of nearly perfect interactive COVID-19 DNA methylation biomarkers that can be used in diagnoses; (2) unlike much other research, this paper advanced the exploration of sites of interaction relationships based on competing risk models; (3) an indication of significant differences in DNA methylation data in identifying critical sites; (4) an indication of cg16279999 (*TESC*), cg00324510 (*PACS1*), cg08949406 (*FHIT*), cg16785077 (*MX1*), cg24002003 (*CHSY1*), cg25932713, and cg22930808 (*PARP9*) as potential druggable targets, and that genes *MND1*, *CDC6*, *ZNF282*, *ATP6V1B2*, *IFI27*, *GCKR*, *PTAFR*, and *CCNI* at the genomic level become potential druggable targets; (5) different from the literature, this paper found that the initial SARS-CoV-2 is hypothesized as a DNA virus with a long incubation period; (6) this new work, together with the existing work in the literature, systematically and accurately describe both SARS-CoV-2 and COVID-19 at the genetic level [8,9,10,11,24].

## 2. Method

This work is in the field of computational biology/medicine. The approach is similar at the genomic level to our papers published in *Vaccines*. This paper is focused on epigenetics biomarkers. All experimental recordings are publicly available. We describe our computational method in this section.

In this work, we used the newly proven method of the max-linear competing logistic regression classifier to classify confirmed COVID-19, healthy controls, and other COVID-19-free respiratory diseases. The new method is very different from other classical statistical and modern machine learning methods, e.g., random forests, deep learning models, and support vector machines [11]. In addition, the new method has enhanced the interpretability of results, consistency, and robustness, as shown in the literature on COVID-19 and several types of cancers [8,9,10,11,15,21,22,23].

This section briefly introduces the necessary notations and formulas for self-containing due to the different data structures used in this work. For continuous responses, the literature [25,26,27] has dealt with max-linear computing factor models and max-linear regressions with penalization. The max-logistic classifier has some connections to the logistic polytomous models but with different structures [28,29,30]. This new innovative approach can be classified as either an AI or machine learning algorithm. However, this new approach has an explicit formula and is interpretable.

Suppose $Y_{i}$ is the ith individual patient’s COVID-19 status ($Y_{i}=0$ for COVID-19-free, $Y_{i}=1$ for infected) and $X_{i}^{\left(k\right)}=\left(X_{i1}^{\left(k\right)},X_{i2}^{\left(k\right)},\ldots ,X_{ip}^{\left(k\right)}\right),k=1,\ldots ,K$ are the beta values, with p=865,859 CpG sites in this study. Here, k stands for the kth type of beta values drawn based on K different biological sampling methodologies. Note that most published works set K=1, and hence the superscript (k) can be dropped from the predictors. In this research paper, K=5, as we have three datasets analyzed in Section 3, and in the first dataset, there are other ARIs (Acute Respiratory Infections) patients, and in the second data set, there are MIS-C pediatrics. Using a logit link (or any monotone link function), we can model the risk probability $p_{i}^{\left(k\right)}$ of the ith person’s infection status as:

(1) $$\log\left(\frac{p_{i}^{\left(k\right)}}{1-p_{i}^{\left(k\right)}}\right)=\beta_{0}^{\left(k\right)}+X_{i}^{\left(k\right)}\beta^{\left(k\right)}$$

or alternatively, we write

$$p_{i}^{\left(k\right)}=\frac{exp\;(\beta_{0}^{\left(k\right)}+X_{i}^{\left(k\right)}\beta^{\left(k\right)})}{1+exp\;(\beta_{0}^{\left(k\right)}+X_{i}^{\left(k\right)}\beta^{\left(k\right)})}$$

where $\beta_{0}^{\left(k\right)}$ is an intercept, $X_{i}^{\left(k\right)}$ is a 1×p observed vector, and $\beta^{\left(k\right)}$ is a p×1 coefficient vector which characterizes the contribution of each predictor (CpG site, in this study) to the risk.

Considering that there have been many variants of SARS-CoV-2 and multiple symptoms (subtypes) of COVID-19 diseases, it is natural to assume that the epigenetic structures of all subtypes can be different. Suppose that all subtypes of SARS-CoV-2 may be related to G groups of CpG sites:

(2) $$\Phi_{ij}^{\left(k\right)}=\left(X_{i,j_{1}}^{\left(k\right)},X_{i,j_{2}}^{\left(k\right)},\ldots ,X_{i,j_{g_{j}}}^{\left(k\right)}\right),j=1,\ldots ,G,g_{j}\geq 0,k=1,\ldots ,K$$

where i is the ith individual in the sample, and $g_{j}$ is the number of CpG sites in the jth group.

The competing (risk) factor classifier is defined as:

(3) $$log\;(\frac{p_{i}^{\left(k\right)}}{1-p_{i}^{\left(k\right)}})=max(\beta_{01}^{\left(k\right)}+\Phi_{i1}^{\left(k\right)}\beta_{1}^{\left(k\right)},\beta_{02}^{\left(k\right)}+\Phi_{i2}^{\left(k\right)}\beta_{2}^{\left(k\right)},\ldots ,\beta_{0G}^{\left(k\right)}+\Phi_{iG}^{\left(k\right)}\beta_{G}^{\left(k\right)})$$

where $\beta_{0j}^{\left(k\right)}$s are intercepts, $\Phi_{ij}^{\left(k\right)}$ is a $1\times g_{j}$ observed vector, and $\beta_{j}^{\left(k\right)}$ is a $g_{j}\times 1$ coefficient vector which characterizes the contribution of each predictor in the j group to the risk.

**Remark 1.**  *In (3), $p_{i}^{\left(k\right)}$ is mainly related to the largest component CF_j_ = $\beta_{0j}^{\left(k\right)}+\Phi_{ij}^{\left(k\right)}\beta_{j}^{\left(k\right)},j=1,\ldots ,G$, i.e., all components compete to take the most significant effect.*

**Remark 2.**  *Taking $\beta_{0j}^{\left(k\right)}=-\infty ,j=2,\ldots ,G$, (3) is reduced to the classical logistic regression, i.e., the classical logistic regression is a special case of the new classifier. Compared with black-box machine learning methods (e.g., random forests, deep learning (convolutional) neural networks (DNN, CNN)) and regression tree methods, each competing risk factor in (3) forms a clear, explicit, and interpretable signature with the selected CpG sites. The number of factors corresponds to the number of signatures, i.e., G. This model can be a bridge between linear models and more advanced machine learning methods (black-box) models. However, (3) retains interpretability, computability, predictability, and stability properties. Note that this remark is similar to Remark 1 in Zhang (2021) [21].*

We have to choose a threshold probability value to decide a patient’s class label in practice. Following the general trend in the literature, we set the threshold to be 0.5. As such, if $p_{i}^{\left(k\right)}\leq 0.5$, the ith individual is classified as being disease-free; otherwise, the individual is classified as having the disease.

With the above-established notations and the idea of a quotient correlation coefficient [31], Zhang (2021) [21] introduced a new machine learning classifier, smallest subset, and smallest number of signatures (S4), for K=1. We extended the S4 classifier from K=1 to K=5 as follows:

(4) (β^,S^,G^)=argminβ,Sj⊂S,j=1,2,…,G{(1+λ1+|Su|)∑k=1K∑i=1n(I(pi(k)≤0.5)I(Yi=1)+I(pi(k)>0.5)I(Yi=0))+λ2(|Su|−Su+G−1Su+1×G−1)}

where I(.) is an indicative function, $p_{i}^{\left(k\right)}$ is defined in Equation (3), S={1,2,…, 865,859} is the index set of all CpG sites, $S_{j}=\{j_{j1},\ldots ,j_{j,g_{j}}\}$, j=1,…,G are index sets corresponding to (2), $S_{u}$ is the union of $\left\{S_{j},j=1,\ldots ,G\right\}$, $\left|S_{u}\right|$ is the number of elements in $S_{u}$, $\lambda_{1}\geq 0$ and $\lambda_{2}\geq 0$ are penalty parameters, and S^={jj1,…,jj,gj,j=1,…,G^} and G^ are the final CpG set selected in the final classifiers and the number of final signatures.

**Remark 3.**  *When the S4 classifier leads to 100% accuracy, the bioequivalence and DNA methylation geometry space can be established, which is a unique property established in (4) that does not appear in other classifiers in the literature [9]. We further note that even with biased samples, e.g., noises, if the performance is perfect, it is an indication of the method being powerful.*

**Remark 4.**  *The case of K=1 corresponds to the classifier introduced in Zhang (2021) [21]. The case of K=1 and $\lambda_{2}=0$ corresponds to the classifier introduced in Zhang (2021) [8].*

The data studied are publicly available. The inclusion and exclusion criteria of the cohorts used in this study are the following: the sample sizes are larger than 20, and the numbers of CpG sites are matched by solving Equation (4). For clinical inclusion and exclusion criteria, readers are referred to the original study designs [3,14,32,33].

Four COVID-19 datasets to be analyzed in this study are publicly available: GSE174818 [14], GSE193879 [3], GSE179325 [32], and GSE219037 [33]. All four datasets used the same platform, GPL21145 Infinium MethylationEPIC, and about 865,859 methylation sites from whole blood samples.

GSE174818 contained peripheral blood samples from 102 COVID-19 patients compared to 26 non-COVID-19 patients. In the second dataset GSE193879, peripheral blood samples were recruited from 43 confirmed MIS-C patients, and 69 non-COVID-19 and 15 COVID-19 pediatric samples were obtained. The third dataset GSE179325 was a cohort of whole blood genome-wide DNA methylation profiling from 473 RT-PCR-positive and 101 RT-PCR-negative SARS-CoV-2 individuals. The fourth dataset GSE219037 is a methylation assay that was performed on samples collected from 252 subjects at different time points as part of the initial SARS-CoV-2 outbreak and later surveillance on the Marine recruits. The data information is summarized in the following Table 1.

## 3. Results and Interpretations

### 3.1. Separability between COVID-19 Patients and Non-COVID-19 Patients Presenting with Respiratory Symptoms

Following the Monte Carlo computational procedure described in the earlier work [22], from 865,859 methylation sites, we identified eight sites (cg16279999, cg24002522, cg00324510, cg08949406, cg16785077, cg23933458, cg24002003, and cg24760467) with cg23933458 corresponding to one IncRNA gene LINC00456 to lead to 96.15% accuracy in classifying COVID-19 patients and non-COVID-19 patients into their respective groups in the first dataset GSE174818. Using the beta values calculated from M/(M + U) and Equations (1)–(4), we obtained the coefficients in Equation (4). Table 2 lists the classifiers and the coefficients of the sites.

In the table, the classifier CF1 in Equation (3) is defined as

−31.725 + 54.1087 × cg24002522 − 24.6712 × cg24002003 + 4.6904 × cg24760467

Then, 0.5 is the threshold for computing risk probability in the max-logistic regression function. Other classifiers are defined similarly. CFmax is defined as the max(CF1,CF2). We note that the threshold 0.5 can be changed to any other values between 0 and 1, and the conclusions will not be changed.

The genes regulated by these CpG sites are listed in the Table 3 as follows.

The information in Table 3 is listed at genecards.org (accessed on 22 November 2022).

Note that three CpG sites (cg00324510, cg08949406, and cg23933458) are not part of the two individual classifiers. Instead, they will be used in Table 4 in Section 3.4. We further note that in this COVID-19 example, the individual classifiers are only CF1 and CF2, and only five sites are used. In the earlier work [8,9,10,15,21], the authors discussed their modeling strategy to avoid overfitting the data. Using extensive Monte Carlo simulation computation, we found that increasing the number of individual classifiers, in this case, will not improve the accuracy and interpretability, and we adopted the final fitted model in Table 2.

### 3.2. CpG Site–Site Interactions

**Figure 1:**
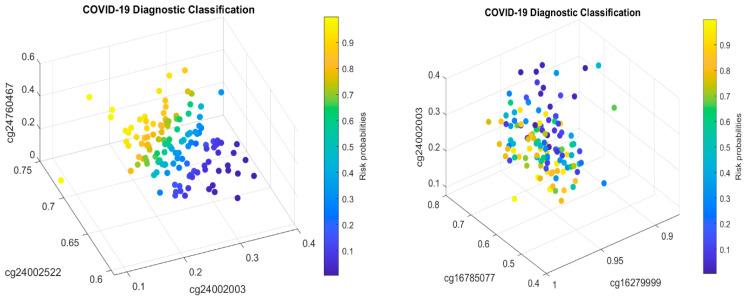
COVID-19 classifiers in Table 2: Visualization of site–site relationships and site risk probabilities. Note that 0.5 is the probability threshold.

We will now explain the CpG site–site interactions. We have two combinations (two competing classifiers): CF1 and CF2. In CF1, three CpG sites (cg24002522, cg24002003, and cg24760467) form a combination (signature, see also Figure 1) with the coefficient signs of the two sites being positive while the third one is negative. In CF2, three CpG sites (cg16279999, cg16785077, and cg24002003) form a different combination (signature), with the coefficient signs of the two sites being negative and the other being positive. Taking cg24002003 as an example, its coefficient strengths (−24.6712 vs. −12.7104) depend on which combination this site falls into, i.e., how it interacts with other sites. Let us consider a basketball team as an analogy. These five CpG sites correspond to five basketball players on a team. The team has two main teammate combinations for scoring. A positive coefficient associated with a player in a teammate scoring combination means that the longer the ball-controlling time of the player, the higher chance of the team to score, and a negative coefficient associated with a player means that the shorter the ball-controlling time of the player, the higher chance of the team to score. In the meantime, which scoring combination is going to score? Under some scenarios, only one combination can score; and under some scenarios, any combination can score. Figure 1 shows such phenomena. There are site–site interactions between competing factors (CF1, CF2), e.g., through cg24002003.

In the two sub-figures, we clearly see two different structures (signature patterns) and the way in which patients are classified into subgroups based on individual beta values. In Section 4.1, we further illustrate the idea of how our classifiers can classify patients into subgroups.

### 3.3. Biological Implications

From Table 2, we can see that CF2 (accuracy 89.06%) performs better than CF1 (accuracy 67.97%). The negative coefficients reveal that the higher the rates of the methylations of cg16786077 and cg24002003, the lower the risk of a patient being COVID-19 positive. The lower the methylations of cg1627999, cg24002522, and cg24760467, the lower the risk of a patient being COVID-19 positive. These observations reveal that regulations of these sites’ methylation rates (beta values) can help prevent a patient from being infected with SARS-CoV-2 and becoming COVID-19 positive.

### 3.4. Cohort-to-Cohort Cross-Validation: Separability between Pediatric COVID-19 Cases and Healthy Controls

The results obtained in Table 2 need to be cross-validated by performing cross-validation (CV). One way is to split the dataset GSE174818 into two sets (modeling set and testing set) and complete the classical CV method. We note that the classical CV has limitations given the heterogeneous population characteristics; see the discussions in later sections. In this paper, we adopted a cohort-to-cohort cross-validation method using a different dataset, GSE193879, to validate the identified CpG sites in Table 2.

Pediatric COVID-19 cases have drawn attention due to vaccine applicability and these patients’ underdeveloped immune systems. This section studies multisystem inflammatory syndrome in children (MIS-C) with or without SARS-CoV-2 infection [3]. For our purposes, we first only targeted patients with confirmed COVID-19 and healthy controls, i.e., we did not include the MIS-C status but focused on 69 non-COVID-19 pediatric samples and 15 COVID-19 pediatric samples. Table 4 reports the fitted max-logistic models and parameter coefficients, followed by our interpretations.

**Table 4:** Performance of individual classifiers and combined max-competing classifiers using blood sample dataset GSE193879 to classify COVID-19+ pediatric patients and healthy controls into their respective groups. CF1 and CF2 are two different classifiers. CFmax = max(CF1–2) is the combined max-competing classifier. The numbers are fitted coefficient values.

Sites	Gene	CF1	CF2	CFmax
	Intercept	35.11	−7.1311	
cg16279999	*TESC*			
cg24002522	*ALCAM*	−42.2336		
cg00324510	*PACS1*		22.369	
cg08949406	*FHIT*	22.441		
cg16785077	*MX1*	−29.4016		
cg23933458	*LINC00456*		10.858	
cg24002003	*CHSY1*		−56.1248	
cg24760467	*LZTS2*			
Accuracy	%	90.48	91.67	92.86
Sensitivity	%	73.33	66.67	100.00
Specificity	%	94.20	97.10	91.30

It can be seen that CF1 values in Table 4 and those in Table 2 have different combinations of sites, i.e., their site–site interactions are different between children and adults. Note that the controls in Table 4 were healthy individuals while the control in Table 2 was other types of respiratory diseases, which makes the comparison not direct. Nevertheless, the coefficient signs of cg16785077 and cg24002003 in both tables are all negative, implying that these sites’ higher methylation levels will reduce the patients’ risk of SARS-CoV-2 infection. One significant fact is that the coefficient signs of cg24002522 are reversed in the two tables, which suggests different responses to SARS-CoV-2 infection between adults and children and different site–site interactions depending on which sites are combined in the classifier (team). As a result, such a phenomenon at the DNA methylation level should be well-understood when applying vaccines to children.

Note that cg23933458 corresponds to the *LINC00456* gene—Long Intergenic Non-Protein Coding RNA (lncRNAs) 456. It can be seen that the larger the beta values of the *LINC00456* gene, the higher the risk of the patient being COVID-19+.

**Figure 2:**
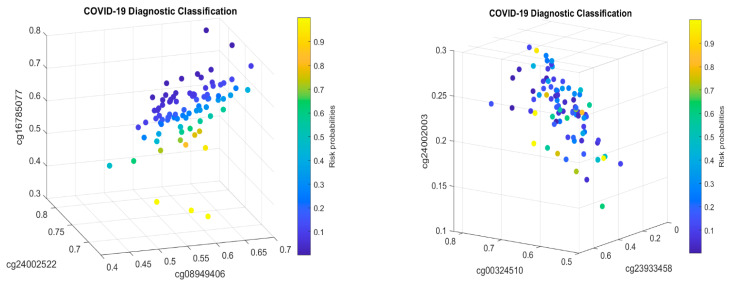
COVID-19 classifiers in Table 4: Visualization of site–site relationships and site risk probabilities. Note that 0.5 is the probability threshold.

Figure 2 presents critical site methylation levels and risk probabilities corresponding to different combinations in the second dataset and Table 4. Like Figure 1, it can be seen that each plot shows a methylation signature pattern and the functional effects of the sites involved.

With the strong performance of eight sites (identified in the first and second datasets), the two datasets serve as a natural double validation of the findings from both datasets. We want to point out that such a kind of validation is different from the cross-validation commonly applied in statistical analysis and experimental validations. We argue that the current experimental technology may not be advanced enough to validate gene–gene and site–site interactions. Also, all experiments applied to animals may not be applicable to humans. In the earlier work [10], we discussed that traditional statistical cross-validation is not valid for heterogeneous population clustering and classifications. In the earlier work [11,15,21,22,23], we found that the findings are intrinsic at the genomic level. They can easily pass cross-validations from cohort to cohort. For example, in the study of genomic biomarker heterogeneities between SARS-CoV-2 and COVID-19, genes (*ABCB6*, *KIAA1614*, *MND1*, *SMG1*, *RIPK3*, *CDC6*, *ZNF282*, *CEP72*, *ATP6V1B2*, *IFI27*, *BTN3A1*, *SERTAD4*, and *EPSTI1*) had robust and nearly perfect performance among 15 cohorts (including different ethics, SARS-CoV-2 subvariants, e.g., omicron, breakthrough infections) with thousands of samples [10], which further led to a confirmation of *MND1*, *CDC6*, *ZNF282*, *ATP6V1B2*, and *IFI27* being meaningful for discovering vaccine adverse effects among COVID-19-convalescent octogenarians [11]. Using this evidence as indirect support, it may be safe to say that the eight sites are optimal DNA methylation biomarkers due to their intrinsic features of site–site interactions, competing interactions of sites–subgroups, and, more importantly, their high-performance accuracy.

### 3.5. Separability between Asymptomatic Cases and Healthy Controls

The CpGs identified in Table 2 were based on hospitalized COVID-19 patients. In this section, we study the performance of the eight CpGs on asymptomatic cases. Using a recent public dataset GSE219037 [33], we obtained the following in Table 5.

We can immediately notice that the performance (accuracy, sensitivity, and specificity) in Table 5 is similar to those in Table 2 and Table 4. The CpGs (cg08949406, cg16785077, and cg23933458) in CF1 in Table 4 and Table 5 have the same coefficient signs, respectively. The CpGs (cg23933458, cg24002003) in CF2 in Table 4 and Table 5 have the same coefficient signs, respectively. We note that the samples related to GSE219037 were a methylation assay performed on samples collected from 252 subjects at different time points as part of the initial SARS-CoV-2 outbreak and later surveillance on the Marine recruits (age 19.77 ± 2.45 years) [33]. It was a longitudinal study. We extracted the asymptomatic cases (76 cases) and healthy controls (96 cases) for the purpose of our study. Putting Table 2, Table 4 and Table 5 together offers a comprehensive understanding of COVID-19 severity and age effects. Based on these observations, we can infer that these eight CpGs are reliable biomarkers for COVID-19 biological studies.

### 3.6. Separability between MIS-C Patients and COVID-19+ Patients

MIS-C is a rare but serious condition associated with COVID-19, which motivates the study of their connection to DNA methylations. We now use the eight sites in Table 2 to study MIS-C patients and COVID-19+ patients. Table 6 lists the fitted results.

In Table 6, the sensitivity corresponds to MIS-C patients, while the specificity corresponds to COVID-19+ patients. It can be seen that the higher the beta values (methylation rates) of cg16785077, cg00324510, and cg23933458, the more severe the MIS-C. The site cg24002003 can be beneficial and harmful to MIS-C, depending on how it interacts with other sites.

**Figure 3:**
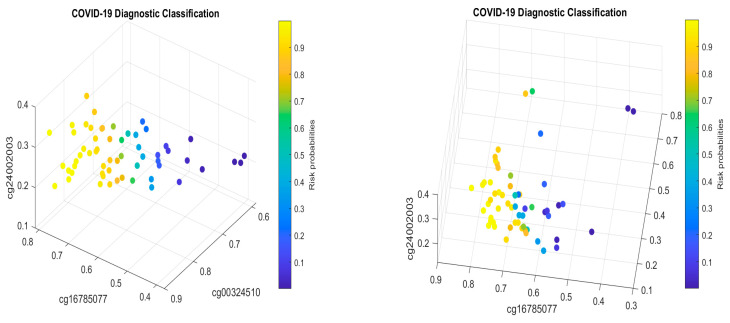
COVID-19 classifiers in Table 6: Visualization of site–site relationships and site risk probabilities. Note that 0.5 is the probability threshold.

Figure 3 demonstrates the signature patterns of CF1 and CF2 in Table 6.

Note that in Table 6, only four CpG sites are used to separate MIS-C from COVID-19+ pediatrics. Nevertheless, all three tables show the strong performance of the identified CpGs.

### 3.7. Cohort-to-Cohort Cross-Validation: Separability among COVID-19 Severe and Mild Cases and Healthy Controls

We used another dataset GSE179325 to validate the identified CpG sites in Table 2.

The dataset GSE179325 contained 113 severe COVID-19+, 360 mild COVID-19+, and 101 healthy control patients. Here we study four classification problems: (1) COVID-19+ vs. healthy; (2) severe COVID-19+ vs. healthy; (3) mild COVID-19+ vs. healthy; (4) severe COVID-19+ vs. mild COVID-19+. They will be presented sequentially in the following sub-sections.

**Figure 4:**
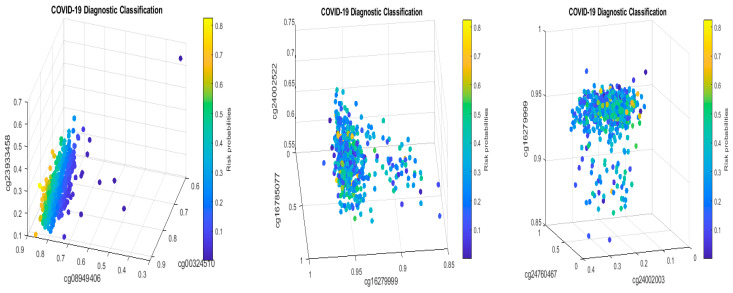
COVID-19 classifiers in Table 7: Visualization of site–site relationships and site risk probabilities. Note that 0.5 is the probability threshold.

For the first case (1), the performance results are listed in Table 7. Figure 4 plots the signature patterns.

**Figure 5:**
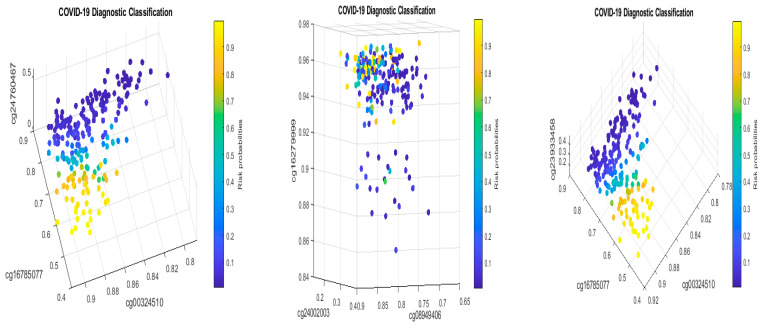
COVID-19 classifiers in Table 8: Visualization of site–site relationships and site risk probabilities. Note that 0.5 is the probability threshold.

For the case (2), the performance results are listed in Table 8. Figure 5 plots the signature patterns.

**Figure 6:**
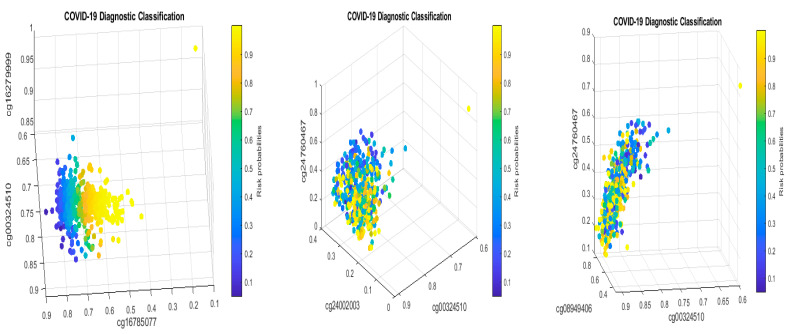
COVID-19 classifiers in Table 9: Visualization of site–site relationships and site risk probabilities. Note that 0.5 is the probability threshold.

For the case (3), the performance results are listed in Table 9. Figure 6 plots the signature patterns.

**Figure 7:**
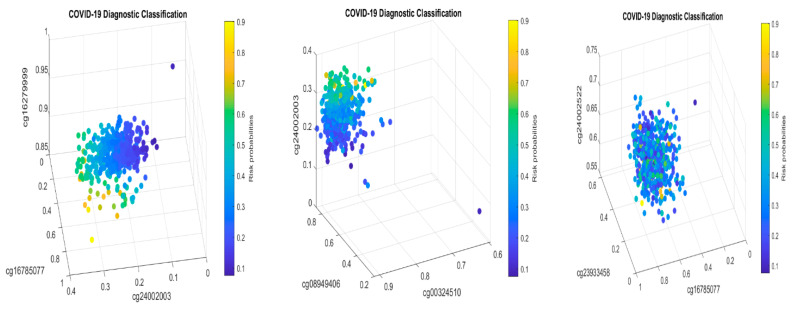
COVID-19 classifiers in Table 10: Visualization of site–site relationships and site risk probabilities. Note that 0.5 is the probability threshold.

For the case (4), the performance results are listed in Table 10. Figure 7 plots the signature patterns.

Interpretations of the results: Comparing Table 7, Table 8 and Table 9 with Table 2, Table 4 and Table 5, and Figure 4, Figure 5 and Figure 6 with Figure 1 and Figure 2, we see different combinations and signature patterns. Although the platforms used the same GPL21145 Infinium MethylationEPIC platform, there are other factors (sex, age, epidemiological and clinical variables, diet, lifestyle, etc.) among these three cohorts. Without considering these factors as confounding variables, the identified eight CpG sites have led to a nearly perfect performance in Table 2, Table 4 and Table 5, which indicate that the eight CpG sites can be used as intrinsic variables, and other factors can be treated as extrinsic variables. When intrinsic variables lead to a nearly perfect performance, the extrinsic variables can provide little additional useful information, i.e., they will not improve the classification accuracy. Based on these observations, in this paper, we only used the eight CpG sites in this section. Table 7, Table 8 and Table 9 show a convincing performance. Nevertheless, in general, including extrinsic variables in GSE179325 may further improve the accuracy in Table 7, Table 8 and Table 9, which is worth further investigation. This step is not essential for identifying critical CpG sites in this paper as the results obtained have proven to be excellent, and we leave it for future work.

Biological implications: We first note that the coefficient signs of cg16785077 and cg24002003 are all negative in Table 7, Table 8 and Table 9 and are consistent with those in Table 2, Table 4 and Table 5. On the other hand, the coefficient signs of cg16279999 are all positive in Table 2, Table 7, Table 8 and Table 9, which indicates that the higher the beta values of this CpG site, the higher the risk of the patient being COVID-19+. In addition, the coefficient signs of cg00324510 and cg08949406 are all positive in Table 4, Table 7, Table 8 and Table 9, which indicates that the higher the beta values of these two CpG sites, the higher the risk of the patient being COVID-19+. Such a phenomenon clearly proves two basic claims: (1) these eight CpG sites and their signature patterns are robust and reliable DNA methylation biomarkers in studying COVID-19 infection; (2) cg16785077, cg24002003, cg16279999, cg00324510, cg08949406, and their regulated genes are potential druggable targets. Here, druggable targets are based on the fact that the reversible and malleable nature of DNA methylation makes it a potential therapeutic target and marker for risk stratification.

Next, we discuss the performance summarized in Table 7, Table 8 and Table 9. Table 8 (severe COVID-19+ vs. healthy controls) shows that these eight CpG sites lead to an overall accuracy of 90.19%, a sensitivity of 94.69%, and a specificity of 85.15%. Such a high performance again shows that these CpGs are reliable DNA methylation biomarkers and robust among different heterogeneous cohorts. Table 9 (mild COVID-19+ vs. healthy controls) shows an overall accuracy of 80.26%, a sensitivity of 80.83%, and a specificity of 78.72%, which is also a satisfactory performance at the DNA level as mildly symptomatic cases and healthy controls can have more similarities in their methylation measures. On the other hand, such a performance is already better than many reported results in the literature. Table 7 (severe and mild COVID-19+ vs. healthy controls) shows an overall accuracy of 79.79%, a sensitivity of 78.86%, and a specificity of 84.16%, which is close to the performance in Table 9. Table 8 and Table 9 together indicate that these eight CpG sites are critical in fighting against COVID-19.

Note that we used eight CpGs in Table 2 to evaluate their performance in Table 4, Table 5, Table 6, Table 7, Table 8 and Table 9 as a cohort-to-cohort cross-validation. Such new types of validations can hardly be found in the literature. The superb performances in all cases lead us to safely infer that the eight CpGs are reliable biomarkers.

### 3.8. Influenza and SSPE Indicated by CpG Site cg16785077 (MX1)

This section discusses the diseases associated with MX1: influenza and subacute sclerosing panencephalitis (SSPE) and their possible connections to other genes.

From Table 10 and Figure 7, we see that severe COVID-19+ and mild COVID-19+ patients can share some common DNA methylation signature patterns, and as a result, it is not easy to separate them. Nonetheless, the eight CpG sites still perform strongly, i.e., they are informative to COVID-19 infection. The coefficient signs of cg16785077 are negative, which coincides with Table 2, Table 4, Table 5, Table 6, Table 7, Table 8 and Table 9. This observation shows that cg16785077 (*MX1*) regulates the gene expression of gene *MX1*, and the higher the methylation rate, the lower the risk of a patient being COVID-19+ or the lower the severity of COVID-19 symptoms. Interestingly, we see that the coefficient signs of cg24002003 (*CHSY1*) are positive in Table 10, which is different from the corresponding coefficient signs in Table 2, Table 4, Table 5, Table 6, Table 7, Table 8 and Table 9. This observation shows that there is a point of change for cg24002003 in terms of how its methylation rate reflects the disease severity, and this phenomenon again shows that site–site interactions should be the key for studying the COVID-19 DNA methylation signature patterns.

It is commonly known that COVID-19 infections share similar symptoms with influenza, especially after omicron infections. In the earlier work [10], the set of genes (*ATP6V1B2*, *IFI27*, *BTN3A1*, *SERTAD4*, and *EPSTI1*) was identified at the genomic level as a set of SARS-CoV-2 biomarkers with nearly perfect performance, based on NP/OP swab PCR samples. Among these five genes, *IFI27* has been studied in the literature, which states that IFI27 discriminates between influenza and bacteria in patients with suspected respiratory infections [34]. Jointly considering our new results at DNA methylation levels and the earlier discovery of *IFI27* in COVID-19 infections, it may be safe to infer that the CpG site cg16785077 (*MX1*) played a role in SARS-CoV-2 transmissions and led to influenza-like symptoms through *IFI27* (NP/OP). However, such a hypothesis can take years to verify.

As to *ATP6V1B2*, it is reported in the literature that de novo mutation in *ATP6V1B2* impairs lysosome acidification and causes dominant deafness–onychodystrophy syndrome [35]. Other than this, it is not known how *ATP6V1B2* affects the brain. SSPE is a progressive neurological disorder in children and young adults that affects the central nervous system (CNS). It is a slow and persistent viral infection related to measles with an incubation period of up to six to eight years. Based on such observations, serious precautions have to be taken, and it can be inferred that there is a likelihood that the CpG site cg16785077 (*MX1*) may lead to SSPE symptoms through the gene *ATP6V1B2*. Likewise, such a hypothesis can take years to verify.

## 4. Other Perspectives

Section 3 applied S4 classifiers (4) to DNA methylation beta values calculated based on the M/(M + U) formula. In this section, we present two alternative approaches to generate DNA methylation beta values and use them to identify CpG sites and evaluate the performance of these sites. We only worked on the dataset GSE174818 as the data made methylated intensities and unmethylated intensities available.

### 4.1. Beta Values Calculated Using (M + 1)/(M + U + 2)

Solving S4 classifiers (4), we obtained CpGs (cg03870777, cg16279999, cg15528722, cg24596788, cg11186858, cg24002522, cg22930808, cg25932713, and cg26301516) to lead 100% accuracies in five different combined classifiers, which are presented in the following Table 11a–e.

The genes regulated by these CpG sites are listed in the following Table 12.

The information in Table 12 is listed at genecards.org (accessed on 22 November 2022).

We note that *PARP9* was linked to SARS-CoV-1 and MERS-CoV in the literature [18,19,20,36,37]. It has also been extensively studied in its connection to SARS-CoV-2 [5,38,39,40,41,42]. In particular, cg22930808 is ranked as the first in [42]. However, in this work, *PARP9*’s functional effects on COVID-19 are through its interaction with other genes (sites) and its interaction with the subgroups. We further note that *PARP9* is not the whole story of the COVID-19 disease, as its intrinsic classifier only has 68.75% accuracy. As a result, the earlier literature research on *PARP9* should be extended. More detailed discussions will be given in what follows.

At the DNA methylation level, our work is the first to identify a set of eight sites (cg03870777, cg15528722, cg24596788, cg25932713, cg16279999, cg11186858, cg24002522, and cg26301516) from 865,859 sites to lead to a perfect 100% accuracy, and as a result, they can be treated as optimum DNA methylation biomarkers. It is a known fact in biological and medical research that finding reliable markers is challenging, mainly due to the limitations of study methods and tools. The proven max-logistic competing models have made such a task possible.

Given that the performance of these sites is based on their interactions, traditional research conclusions/recommendations from individual site analysis, including pathway analysis, can be doubtful as long as those results cannot be verified to lead to accurate results.

A natural question is whether other sites can lead to 100% accuracy. We have found that a set of nine sites (cg03870777, cg16279999, cg15975806, cg24596788, cg11186858, cg24002522, cg25932713, cg11036672, and cg26301516) lead to 100% accuracy. It can be expected there will be many such combinations as long as we are allowed to include more sites. For this reason, we only report the smallest number of sites in Table 11a–e.

Given that *PARP9* was linked to SARS-CoV-1, several research papers have discovered its potential functions in SARS-CoV-2, e.g., as a noncanonical RNA sensor for RNA viruses in initiating and amplifying protective immunity [41]; it is worth jointly studying *PARP9* together with *FNDC3B* and a pseudogene *LOC100422212*.

Besides *PARP9*, the three genes *KRT8*, *SEC14L1*, and *ALCAM* have also been linked to COVID-19. In particular, *KRT8* (cg03870777) is a marker of DATP representative immunofluorescence staining for pro-SPC, *KRT8*, and DAPI in both control and COVID-19 lung tissue [6]. *SEC14L1* is anti-inflammatory, which is significantly downregulated in COVID-19 patients [43]. On the other hand, the percentage of monocytes (CD14+) expressing the cell adhesion molecule *ALCAM* is strongly upregulated in SARS-CoV-2+ patients [44].

Among all three component classifiers CF1, CF2, and CF3 in Table 11a, *KRT8* is a component of CF1, both *SEC14L1* and *ALCAM* are components of CF2, while *PARP9* is a component of CF3. These facts reveal that these genes only characterize partial information about COVID-19 and must be combined with other genes to take functional effects. As a result, any research conclusions made without studying site–site interactions can be incomplete, and their usefulness can be genuinely doubtful. An analogy is that studying trees cannot solve the problem of the forest.

**Figure 8:**
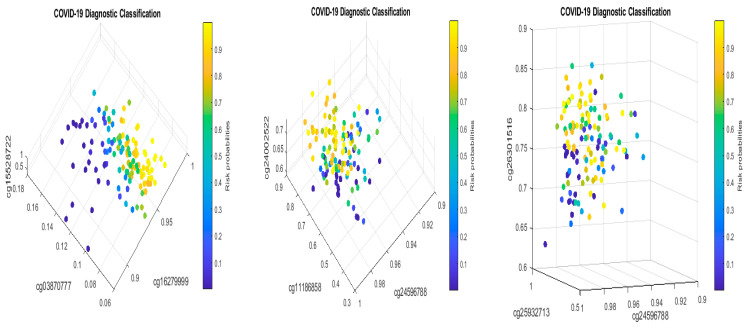
COVID-19 classifiers in Table 11a: Visualization of site–site relationships and site risk probabilities. Note that 0.5 is the probability threshold.

Figure 8 presents critical site methylation levels and risk probabilities corresponding to different combinations in Table 11a. It can be seen that each plot shows a methylation signature pattern and functional effects of the sites/genes involved.

Biological implications: From Table 11a and Figure 8, we can see that increasing the methylation levels of cg03870777, cg15528722, cg24596788, and cg25932713, and decreasing the methylation levels of cg16279999, cg11186858, cg24002522, and cg26301516, will lower the risk of contracting COVID-19 disease. In particular, cg25932713 (*PARP9*) with higher methylation levels will benefit an individual.

We note that this set of genes (*KRT8*, *TESC*, *TTC33*, *LOC100422212*, *SEC14L1*, *ALCAM*, *PARP9*, and *FNDC3B*) does not overlap with the set of genes (*ABCB6*, *KIAA1614*, *MND1*, *SMG1*, *RIPK3*, *CDC6*, *ZNF282*, and *CEP72*) identified at the genomic level as a set of COVID-19 biomarkers with optimum performance, based on whole blood samples [8,9,10]. Furthermore, these genes also do not overlap with the set of genes (*ATP6V1B2*, *IFI27*, *BTN3A1*, *SERTAD4*, and *EPSTI1*) identified at the genomic level as a set of SARS-CoV-2 biomarkers with nearly perfect performance, based on NP/OP swab PCR samples [10]. Such biomarker heterogeneities can be explained as they represent different levels, i.e., the COVID-19 DNA methylation level, COVID-19 genomic level, and SARS-CoV-2 genomic level. Putting them all together establishes a uniformly integrated and comprehensive understanding of the infection and disease and hence can benefit the development of vaccines, antiviral drugs, and medical treatments. Based on our limited knowledge, scientists have not figured out how the viruses were formed and how they were transmitted from one to another [1,2,3,4,5,6,7]. Our work certainly provides a completely new direction (initial virus being a transcribed viral DNA into RNA virus) to explore.

**Figure 9:**
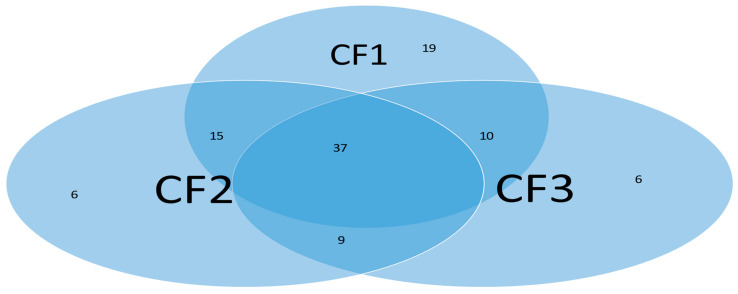
Venn diagram of variants of COVID-19 (the first dataset).

Subgroups classified by classifiers: Using the probability threshold of 0.5, we can further divide patients into subgroups. Clearly, patients in the same subgroup share basic intrinsic disease medical information. Figure 9 is a Venn diagram illustrating each classifier’s performance. There are a total of seven subgroups. In the Venn diagram, those patients who fall in the intersections are relatively easy to be tested and confirm as positive, while for those who only fall in one category, it is relatively hard to test and confirm their status. Three individual classifiers can be explained as having three COVID-19 tests using three different testing procedures (kits), and with the three tests being positive, the probability of infection will be higher depending on the sensitivity and the specificity of each test.

We can see in Figure 9 that about 37% (37 out of 102) of the COVID-19 patients were confirmed by every individual classifier, i.e., all eight sites and their interactions took effect. The status of 37% of patients is more complicated than that of other patients in the cohort. This Venn diagram clearly reveals any individual site alone will not be an efficient targeting point. Therefore, we have to study the site–site interactions and site–subgroup interactions jointly.

Finally, comparing Table 2 with Table 11a, we see that cg16279999 and cg24002522 appear in both tables, and their coefficient signs are also both positive. Such a phenomenon again indicates that these two sites are essential in the classifications of COVID-19+ patients and patients with other types of respiratory diseases, and lowering their methylations can benefit patients.

### 4.2. Overall Performance and Interpretations

Individual classifiers in Table 11b–e can be interpreted similarly. We summarize the coefficient signs in Table 11a–e in Table 11f.

We can immediately see that, except row cg24002522, all other rows show the same sign of the fitted coefficients within each row. Notice that the CF2 in Table 11c has a sensitivity of 48.04%, which indicates this classifier can only be workable for a specific group of patients and may be replaced by other classifiers.

Changing to a different set of genes and obtaining the similar patterns in Table 11f is very unlikely. Three sites could be adequate enough if we sought a lower accuracy of less than 89.84%. For example, CF1 in Table 11a, and CF1 and CF2 in Table 11b are each adequate enough. We also note that many published works have not paid attention to the specific features of the fitted models and, hence, can be less informative. With the uniform patterns in Table 11f the methylation sites identified in the table are indeed meaningful.

The three individual classifiers can also be interpreted as follows. As mentioned earlier, each classifier is a test kit. As long as any of the three tests results in an individual being positive, the individual is COVID-19 positive with 100% probability given the specificity has been 100% in practice. Each table corresponds to a test kit manufacturer; five manufacturers use different technological combinations.

We now use CF3 in Table 11a, CF2 in Table 11b, and CF1 in Table 11c to interpret site–site interactions and their effects. First, note that these three classifiers share the same genes (*LOC100422212, PARP9, FNDC3B*), but the sites are different: cg24596788, cg25932713, and cg26301516; cg24596788, cg22930808, and cg26301516; and cg24596788, cg22930808, and cg26301516 in CF3, CF2, and CF1, respectively, in their corresponding tables. One immediate question is why CF2 (Table 11b) and CF1 (Table 11a) are fitted differently, even though the site combinations are the same. This phenomenon is due to the objective function in Equation (4) being locally flat in many regions, where different solutions can lead to the same precision when combined with other classifiers. Of course, CF2 (Table 11b) can be mathematically replaced by CF1 (Table 11c). Second, CF3 (Table 11a) underperformed the other two classifiers in Table 11b,c due to the use of different PARP9 sites. We may think that cg22930808 (PARP9) is more informative than cg25932713 (PARP9) based on the performance among the above classifiers. Looking at CF3 in Table 11b, we can see that the performance is reversed, i.e., which sites are being combined. This phenomenon tells us that at the DNA methylation level, we need to pay attention to the site information; simply listing a gene name is not enough. These three classifiers reveal that there are site–site interaction effects at the DNA methylation level.

A final note of this section is that we can combine Table 11a–e to form Table 11g, with each individual classifier having the same and highest accuracy of 89.84%.

Note that seven sites (seven genes) are used in Table 11g, while seven sites (six genes) are used in Table 11c.

### 4.3. The Roles of MX1 and PARP9 in SARS-CoV-1 and MERS-CoV

In their paper, Daugherty et al. [18] found that *PARP* genes have a broad role in ADP-ribosylation in host–virus conflicts. Among the 17 human *PARP* genes, *PARP9*, *PARP14*, and *PARP15* are the only three human genes that contain both *PARP* domains and macrodomains. Macrodomains uniquely recognize, and in some cases can reverse, protein mono-ADP-ribosylation, and the authors observed strong signatures of recurrent positive selection throughout the macro-PARP macrodomains. It has been found that the ADP-ribose-10-monophosphatase domains of SARS-CoV and human coronavirus 229E (HCoV-229E) mediate resistance to antiviral interferon responses [36]. It has been found that proteins *PARP9* and *MX1* are shared across SARS-CoV-2–infected PTC and DTC as well as MERS-CoV-infected PTC [20]. Expressions of *MX1* appeared in the targets of the IFN-g pathway in response to viral infection with SARS-CoV-1 and SARS-CoV-2. However, these two genes *MX1* and *PARP9* did not appear in the earlier work [8,9,10,11,24], which tested more than 20 cohorts with thousands of sample patients and reached nearly perfect accuracy. Nevertheless, at the DNA methylation levels, in Table 2, Table 4, Table 5, Table 6, Table 7, Table 8, Table 9 and Table 10, the CpG site cg16785077 (*MX1*) appeared to be the most significant one, as well as *PARP9* in Table 11a–f and its connections to SARS-CoV-1 and MERS-CoV, as discussed in the literature. Most recently, SARS-CoV-2 infection and type I interferon-driven inflammation were found to reduce serotonin [45]. With all these observations and the finding that the reverse-transcribed SARS-CoV-2 RNA can integrate into the genome of cultured human cells and can be expressed in patient-derived tissues [16], we can hypothesize the following: (1) the initial SARS-CoV-2 is a DNA virus; (2) SARS-CoV-2 rooted genes *MX1* and *PARP9* at the DNA level had a long incubation period (we note that HIV has a long incubation period up to 20 years). Of course, we should not assume that all the published literature is true. These hypotheses, including those published in the literature, have to be tested.

### 4.4. TQCC Transformed Methylation Performance

In Equation (4), the last part of the formula is related to the so-called tail quotient correlation coefficient (TQCC) introduced in [31]. TQCC is a sample alternative to Pearson’s correlation coefficient, and these two sample-based measures are asymptotically independent, which is a unique, appealing property in application. The values of TQCC are between zero and one. The formula we used in this section is TQCC-beta-value = (*V*/*N* + *N*/*V*)/((*V*/*N* + 1) × (*N*/*V* + 1) − 1) *with V* = *U* + 1 *and N* = *M* + 1.

Solving (4) leads to Table 13.

The genes regulated by these CpG sites are listed in the following Table 14.

The information in Table 14 is listed at genecards.org (accessed on 22 November 2022).

Comparing Table 13 with Table 2 and Table 11a, the thirteen CpG sites that led to 100% accuracy in Table 13 differ from those eight CpG sites in Table 2 and eight CpG sites in Table 11. This phenomenon is caused by how we transform (un)methylated intensities into a value between zero and one. This is the first time we applied a monotone TQCC transformation formula in a DNA methylation study compared with the common approach in the literature of beta-value transformation. Note that in computing the beta-value transformation widely used in the literature, there is no difference between 50/(50 + 50) and 2513/(2513 + 2513), which can be a potential issue of the methodology itself.

The question will be which set of CpG sites in Table 2, Table 11 and Table 13 is more reliable given their high performance (nearly perfect) in classifications. Such a question needs to be further addressed. However, it may take years to figure out as it requires lab experiments to test all the identified sites and their functions, which is beyond the scope of this paper. Instead, we applied the sites in Table 11a to datasets GSE193879 and GSE179325 and found that the overall performance of these eight sites is lower than the performance of the eight sites listed in Table 2. From this observation, in Section 5, we will focus our discussions and conclusions based on those eight sites in Table 2. However, other CpGs in Table 11a–e and Table 13 are worth further consideration and investigation.

### 4.5. Driver Genes for Maturity-Onset Diabetes of the Young

In Section 4.1 and Section 4.2, we demonstrated two different data processing methods and showed that the outcomes were different, although they all led to nearly perfect accuracies. Such results raise urgent scientific questions: (1) whether or not the analytical outcomes are reliable; (2) if they are, which one is the best, i.e., the best of the best; (3) mathematically, whether or not they are equivalent; (4) practically, which one is more preferable by the field.

For (1), the analogy is “All roads lead to Rome”, though some of them take a long time to complete. For (2), we need to set some criteria first and then decide. In the earlier work [21], the authors showed that their method can lead to a miniature set while others may not. For (3), in our earlier work [9], we showed a mathematical equivalence and biological equivalence between different data collection schemes. A mathematical equivalence will be useful for identifying reliable biomarkers while a biological equivalence will be useful for developing antiviral drugs. For (4), it truly depends on the available technology and the knowledge domain. Table 11f clearly shows that our model is reliable and informative.

In addition to DNA methylation analysis, we conduct a new study at the genomic level beyond the earlier COVID-19 research work [8,9,10,11]. We note that the genes identified in this section cannot directly connect to the CpG sites identified in Section 3. This section aims to demonstrate that our model (3)–(4) can outperform AI, ML, and probability algorithms and lead to better and stronger results, which indirectly justify the optimality of our findings in Section 3. We want to note that many published results are useful and insightful; however, the problem comes from the analysis methods researchers applied, i.e., although researchers had excellent research designs, suboptimal results were attained due to the limitation of the analytical approaches used. In this section, we use a real data application to demonstrate what has been missed in the published work.

The etiology of severe forms of COVID-19, especially in young patients, remains a salient unanswered question [46]. The researchers used Integrative AI, ML, and probabilistic programming to distinguish non-critical and critical patients with COVID-19 and identified *ADAM9* as a driver of disease severity and a candidate therapeutic target. A causal network treated *RAB10*, *MCEMP1*, *MS4A4A*, *GCLM*, and *ADAM9* as five putative driver genes. Directly applying our model Equation (4) to these five genes and the dataset GSE172114 [46], we found that our model produced better-performing results than the results reported in the paper [46]. We obtained an overall accuracy of 98.55% with a sensitivity of 100% and a specificity of 95.65%, which demonstrates that these five genes are informative in young COVID-19 patients and that our model (4) has advantages over other models even if the same data is used; as a result, we can conclude that analytical methods matter.

In the earlier work [8,9,10], the five genes (*ABCB6*, *KIAA1614*, *MND1*, *RIPK3*, and *SMG1*) led to 100% accuracy and established the geometry of genomic space. The dataset GSE172114 contains 15,957 genes, much less than the number of genes in datasets we used in the earlier work. GSE172114 does not contain *KIAA1614* and *MND1*, so we cannot test the performance of the five genes (*ABCB6*, *KIAA1614*, *MND1*, *RIPK3*, and *SMG1*) using this dataset GSE172114.

More interestingly, we found from the GSE172114 dataset the gene *GCKR* (Glucokinase Regulator) can be more informative than *ADAM9* as *GCKR* leads to 100% accuracy combined with only four other genes with different combinations: (1) *GCKR*, *PTPN12*, *HNRNPLL*, *RN7SKP80*, and *PRR13*; (2) *GCKR*, *PTPN12*, *HNRNPLL*, *TLK1*, and *RN7SKP80*; (3) *GCKR*, *PAOX*, *NDUFV1*, *RP11-351I24.1*, and *RP4-568C11.4*. Many such combinations will lead to 100% accuracy if five or more genes are allowed. Between (1) and (3), *GCKR* is the common gene, which shows *GCKR* is in the center of gene combinations and interacts with other genes to take effect.

More importantly, as reported at genecards.org (accessed on 22 November 2022), diseases associated with *GCKR* include “fasting plasma glucose level quantitative trait locus 5 and maturity-onset diabetes of the young”, which is a severe issue in the young. We justify our findings in what follows.

Let us consider the three genes *GCKR*, *HNRNPLL*, and *METTL9* and their combinations. The information for the other two genes is listed here. *HNRNPLL* (Heterogeneous Nuclear Ribonucleoprotein L Like) is a protein-coding gene. Among its related pathways is translational control. *METTL9* (Methyltransferase Like 9) is a protein-coding gene. Diseases associated with *METTL9* include autosomal recessive deafness 22.

Table 15 reports the performance of the three genes.

Clearly, we can see that these three genes *GCKR*, *HNRNPLL*, and *MEYYL9* performed as well as the five genes *RAB10*, *MCEMP1*, *MS4A4A*, *GCLM*, and *ADAM9* in the paper [46]. With these observations, the function of *GCKR* demands a deeper understanding of COVID-19 effects in critical young patients.

## 5. Discussion and Conclusions

### 5.1. Discussion

Many COVID-19 research results at the genomic level have been published in the literature. These published results have explored the pathological causes of COVID-19 infection from various aspects. Due to the limitations of research methodology, some of the published results can hardly be cross-validated from cohort to cohort. One exception is that the earlier work [8,9,10] cross-validated thirteen genes across fourteen cohort studies with thousands of patients, heterogeneous ethics, ages, and geographical regions and showed interpretable, reliable, and robust results. The work at the genomic level was a comprehensive study with nearly perfect performance. We did not find any other method that led to 100% accuracy in the literature, not to mention interpretability. In the literature, *MX1* (cg25888371) and *PARP9* (cg22930808) combined with eight other genes (i.e., a total of 10 genes) to reach an overall 78.4% accuracy [42] which is much lower than 89.84% in CF2 in Table 2 with *MX1* (cg16785077) combined with only two other genes (i.e., a total of three genes). In addition, many studies have focused on only a single cohort whose representativeness cannot be assessed.

Many published results have studied the functional effects of genes based on single gene expression value changes. They lack an interaction effects study, mainly due to the limitations of the research methods. Using the cg16785077-regulated gene *MX1* as an example, in CF2 in Table 2, it must be jointly studied with another two genes *TESC* and *CHSY1*, to fully understand its functional effects on COVID-19, as it does not appear in CF1.

Since COVID-19 started in December 2019, many genes have been reported to be linked to various diseases. However, they lack mathematical proof or biological equivalence. They just happened to be significant in one cohort study. For example, SARS-CoV-2 entering the brain [47], COVID-19 vaccines complicating mammograms [48], memory loss and ‘brain fog’ [49], and COVID-19 endothelial dysfunction causing erectile dysfunction [50], amongst other symptoms, have been reported. From our findings, cg16785077 (*MX1*) may provide a clue for brain-related COVID-19 symptoms; cg08949406 (*FHIT*) may lead to an answer about breast cancer-related COVID-19 symptoms; cg24002003 (*CHSY1*) may be informative for temtamy preaxial brachydactyly syndrome- and brachydactyly-related COVID-19 symptoms.

In the literature, the gene *MX1* has been reported to affect our response to COVID-19+ [5,42,51,52,53,54,55,56,57]. *MX1* becomes a potential druggable target [51]. We want to note that *MX1* did not appear in the genomic biomarkers with optimum performance for SARS-CoV-2 and COVID-19, though it is regulated by cg16785077 found in this study. Note that multiple sites can regulate a gene, e.g., *MX1* can be regulated by 188 CpG sites. In addition, from Table 2, we can see that cg16785077 only appears in CF2, i.e., not in CF1, which tells us that there is a larger portion of COVID-19+ which is not caused by or related to cg16785077 (*MX1*).

Recall that the earlier work dealt with genomic biomarkers [8,9,10,24], and identified genes (*ABCB6*, *KIAA1614*, *MND1*, *SMG1*, *RIPK3*, *CDC6*, *ZNF282*, and *CEP72*) at the genomic level as a set of optimally performing interactive COVID-19 biomarkers based on whole blood samples and genes (*ATP6V1B2*, *IFI27*, *BTN3A1*, *SERTAD4*, and *EPSTI1*) at the genomic level as a set of optimally performing interactive SARS-CoV-2 biomarkers based on nasopharyngeal (NP) and oropharyngeal (OP) PCR swab samples. The genes listed in Table 2 in this paper are *TESC*, *ALCAM*, *PACS1*, *FHIT*, *MX1*, *LINC00456*, *CHSY1*, and *LZTS2*, which are biomarkers at the DNA methylation level or epigenetic level and are different from those optimally performing biomarkers at the genomic level or RNA-seq level. We used basketball players on a team to explain the site–site interactions among sites. In the earlier work [9,10], we used the same analogy to interpret gene–gene interactions. Considering the fundamental differences between DNA and RNA in terms of their functions, we can consider, on a basketball team, cg16279999 (*TESC*), cg24002522 (*ALCAM*), cg00324510 (*PACS1*), cg08949406 (*FHIT*), cg16785077 (*MX1*), cg23933458 (*LINC00456*), cg24002003 (*CHSY1*), and cg24760467 (*LZTS2*) as coaches, managers, trainers, and team doctors, while considering *ABCB6*, *KIAA1614*, *MND1*, *SMG1*, *RIPK3*, *CDC6*, *ZNF282*, *CEP72*, *ATP6V1B2*, *IFI27*, *BTN3A1*, *SERTAD4*, and *EPSTI1* as players. Each gene (site) has a specific role on the team, and they interact. This analogy and the analogies used in earlier sections can help some readers understand the model and site–site interactions, though they do not provide insightful biological implications.

At the genomic level, *MX1* may trigger diseases like influenza and subacute sclerosing panencephalitis (SSPE), a progressive neurological disorder in children and young adults that affects the central nervous system (CNS); SSPE can have up to a 6- to 8-year- long incubation period for youngsters, which can be the most significant concern for COVID-19 infection. Therefore, urgent efforts are needed to investigate this potential. In addition, other symptoms listed in Section 3.2 are also urgent.

As to *MX1* becoming a potential druggable target, the site cg16785077 should be the true druggable target. In addition, the genes *MND1*, *CDC6*, *ZNF282*, *ATP6V1B2*, *IFI27*, and *GCKR* at the genomic level become potential druggable targets [10].

A combination of cg16785077 (*MX1*), *ATP6V1B2*, and *IFI27* can explain many COVID-19 infection symptoms, e.g., influenza-like symptoms (omicron symptoms), and ‘brain fog’. However, the connections of reported diseases in the literature to COVID-19 are not confirmed due to the infection, and the diseases may not be relevant. Our findings can certainly provide useful clues. Again, we should not assume all published literature is true. Many published papers are based on correlation studies. Our new study should not be regarded as a correlation study for the following reason: the identified reliable and nearly perfectly performing DNA methylation biomarkers established a mathematical equivalence between the disease and the site–site interactions. A mathematical equivalence can reveal what a result may be, which can be more informative than unreliable and non-proven published causative studies. Furthermore, ‘causal’ implies invariance, and a mathematical equivalence shows that our new results are invariant. In other words, if an invariance cannot be established, the claimed causal relationships are doubtful. Based on these observations, it can be inferred that our findings at the DNA methylation level are mostly closed to the cause of COVID-19 disease regardless of whether or not all the published literature is true.

All the findings reported in this paper are based on experimental results from published work. One may argue that the conclusions from this paper are too strong to believe. Additional experimental validations are needed. The methodology applied in this paper may not be implementable in new experiments as site–site interactions have never been discussed in any biological literature. On the other hand, a question arises: should we believe published results that may not be cross-validated from different cohort studies, though they were based on experiments? We can definitely question many published results from experiments due to the limitation of analysis methods, where as a result, they may be misleading.

Classical logistic regression models have been widely used as a benchmark and baseline in medical data modeling and inference. However, Teng and Zhang [58] have pointed out it has a fundamental flaw as it does not directly specify relative treatment effects in the model, and as a result, misleading results have been reported in the literature. A more robust model, called AbRelaTEs, was introduced in [58], and the classical logistic regression becomes a special case of AbRelaTEs. In scientific research, we first compare the accuracies and then the computational time when applying multiple candidate models. When the accuracies from different models are significantly different, reporting the results from models with lower accuracies becomes meaningless unless we study the properties of the models. In this regard, we do not compare the max-logistic regression with the logistic regression. Interested readers can find many published works using logistic regressions. Section 4.5 demonstrated a real data example where the max-logistic regression outperforms AI algorithms, machine learning, and probabilistic algorithms. Based on these observations, we argue that the max-logistic regression should be considered as a baseline model and a benchmark model in terms of accuracy.

In medical research, another issue is to avoid overfitting the data, and cross-validation has been applied to many studies. However, as pointed out in [9], cross-validations should be applied to homogeneous datasets. When data are collected from heterogenous populations, i.e., subtypes in this paper, regular cross-validations will lead to wrong variable selections and miss the most important critical CpG sites and genes. Nevertheless, suppose one really wants to apply cross-validations to the max-logistic regressions in this dataset, given its specificity being 100% accurate. In that case, any 50–50 split of datasets will still lead to 100% specificity and at least 94% sensitivity. The reason is that the S4 classifier in Equation (4) has been mathematically proved to reach the smallest set of genes [21], the strongest property in the variable selection literature. For this reason, we do not apply the classical cross-validations to our model fitting. Instead, we apply an even more challenging cross-validation procedure: cohort-to-cohort cross-validations. It can be expected that many published works may fail or have very low accuracies when applied to cohort-to-cohort cross-validations. We note that cohort-to-cohort cross-validations are still rather sparse in the literature, and we refer interested readers to the earlier works [10,11,15,21,22,23].

Different from many research papers that considered biological heterogeneity, e.g., the stage of disease, treatment protocol, and inter-individual variation factors as well as demographic and baseline clinical characteristics like ethnicity, age, and sex, as well as containing an absence of pre-pandemic controls and their unknown health status, this paper only focused on DNA methylations CpG sites. In our opinion, those heterogeneities are extrinsic variables; they can provide additional information when studying the severity and duration of the disease. However, the CpG sites are intrinsic factors, and as long as they reach nearly perfect prediction powers, the extrinsic variables can provide little additional information. Interested readers are referred to published work for research on heterogeneity, epigenetic clocks, and surface contamination [59,60].

Many published medical research papers do not report the fitted explicit coefficients and interpret the coefficients and the meaning of their associated positive and negative signs, which leave the results in the dark. We reported and interpreted all fitted coefficients, as in Table 2 and the formula below. We also used different trials and datasets to justify the fitted coefficients. We note that the fitted signs of the coefficients are the most important parts of scientific reasoning. However, many published research papers should have paid more attention to this critical issue and cohort-to-cohort validations.

**Figure 10:**
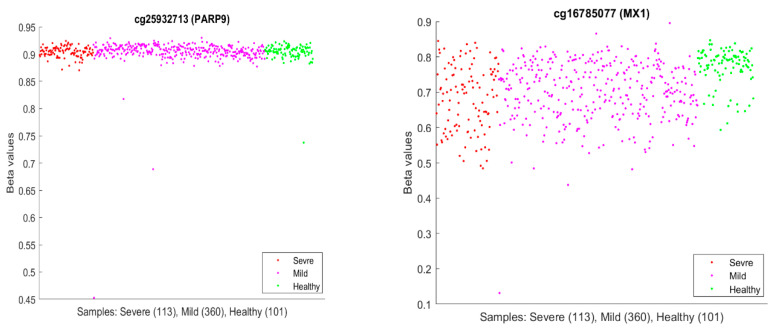
Beta values of cg25932713 (PARP9) and cg16785077 (MX1) in GSE179325.

It is usual to see epigenetic changes [61], including DNA methylation changes, in the host (patient) blood and solid tissue after the infection of DNA/RNA viruses and bacteria, which may be partially attributed to the systemic inflammation brought by the host–virus interaction and anti-infection immunity. In our work, our hypotheses are based on CpG site–site interactions and site–subtype interactions with nearly perfect accuracy. We note that the existing literature has seldom addressed these features and, more importantly, has lacked convincing accuracies. In the literature, we discussed earlier that *PARP9* and MX1 had been linked to COVID-19. Figure 10 plots beta values of cg25932713 (*PARP9*) and cg16785077 (*MX1*).

In Figure 10, individual beta values clearly cannot distinguish severe, mild, and healthy cases. Such a phenomenon again confirmed that CpG site–site interactions are the key to uncovering the truth. We further note that in GSE174818, all patients are hospitalized patients with either COVID-19 or other respiratory diseases. As a result, the CpG sites identified are COVID-19-specific.

In their model of methylation clocks, the authors [33] identified cg26312951 (*MX1*) and cg00959259 (*PARP9*) to be effective. These two CpGs are different from the CpGs in Table 2 and Table 11a. This phenomenon needs further studying.

We have shown that pediatric COVID-19 cases have different DNA methylation signatures from adults. As a result, treatments for children should be evaluated with additional care.

Finally, methylation analysis can provide more comprehensive and detailed information, particularly in understanding the interplay between viral infection and the host genome. Notably, recent studies have reported associations between DNA methylation changes and the worsening of SARS-CoV-2 infection [32,62]. Additionally, this method has shown promise in identifying mild cases [32], predicting outcomes [14,63], and assessing treatment strategies [64], thus offering valuable insights for personalized medicine. The aim of our research was to identify epigenetic changes associated with COVID-19 symptoms or infection status and to explore the causality of the disease.

### 5.2. Conclusions

Our work is the first to study COVID-19 site–site interactions at the epigenetic level. We discovered a miniature set of nearly perfect interactive COVID-19 DNA methylation biomarkers. Unlike much other research, this paper advances the exploration of site interaction relationships based on competing risk models. We indicate significant differences in DNA methylation data in identifying critical CpG sites. We identify cg16279999 (*TESC*), cg00324510 (*PACS1*), cg08949406 (*FHIT*), cg16785077 (*MX1*), cg24002003 (*CHSY1*), cg25932713, and cg22930808 (*PARP9*) as potential diagnostic and druggable targets. In addition, the genes *MND1*, *CDC6*, *ZNF282*, *ATP6V1B2*, *IFI27*, and *GCKR* at the genomic level become potential druggable targets [10]. Here, potential druggable targets mean that these CpG sites are either over-methylated or under-methylated, or their site–site interactions undergo changes, and the genes are either over-expressed or knocked down, or the gene–gene interactions undergo changes, which point toward directions for developing antiviral drugs.

This new work, together with the earlier work [8,9,10,11,24], systematically and accurately describes both SARS-CoV-2 and COVID-19 at the genetic level.

We have discussed that the sites (genes) at the DNA methylation level are different from optimum genomic biomarker genes at the genomic level. We hypothesize that the initial SARS-CoV-2 was a DNA virus and then was transcribed to an RNA virus. Such findings open the door to understanding the pathology of the SARS-CoV-2 infection and COVID-19 disease.

It would be more significant if the interactions between methylation and phosphorylation, ubiquitination, acetylation, and their respective interactions in epigenetics could be studied. It is worth studying how the impact of COVID-19 would change in actively differentiating cells vs. terminally differentiated cells based on the CpG interactions. In addition, pathway analysis merits further study. We leave these aspects for a future project once relevant data can be obtained.

Finally, the most innovative work in this paper are the site–site interactions, gene–gene interactions, and site–gene interactions, which have been missed in published papers that applied other non-interactive variable analysis methods.

## Figures and Tables

**Table 1:** Descriptions of four datasets used in the study.

Data Source	COVID-19 Severity	Control	Size (Positive)	Size (Negative)	Age (Years)
GSE174818 [14]	Hospitalized	Hospitalized other respiratory disease	102	26	21–89
GSE193879 [3]	N/A	Healthy	15	69	0.5–17
GSE179325 [32]	Severe and mild	Healthy	473	101	19–103
GSE219037 [33]	Asymptomatic	Healthy	76	96	18–28

**Table 2:** Performance of individual classifiers and combined max-competing classifiers using blood sample dataset GSE174818 to classify hospitalized COVID-19 patients and other types of patients (as control) into their respective groups. CF1 and CF2 are two different classifiers. CFmax = max(CF1, CF2) is the combined max-competing classifier. The numbers are fitted coefficient values.

Sites	Gene	CF1	CF2	CFmax
	Intercept	−31.725	−40.3148	
cg16279999	*TESC*		65.6839	
cg24002522	*ALCAM*	54.1087		
cg00324510	*PACS1*			
cg08949406	*FHIT*			
cg16785077	*MX1*		−26.8699	
cg23933458	*LINC00456*			
cg24002003	*CHSY1*	−24.6712	−12.7104	
cg24760467	*LZTS2*	4.6904		
Accuracy	%	67.97	89.06	96.88
Sensitivity	%	60.78	88.24	98.04
Specificity	%	96.15	92.31	92.31

**Table 3:** Descriptions of genes in Table 2.

Gene Name	Gene Type	Associated Diseases
*TESC* (Tescalcin)	Protein Coding	Van Den Ende–Gupta Syndrome
*ALCAM* (Activated Leukocyte Cell Adhesion Molecule)	Protein Coding	Stork Bite and Melanoma
*PACS1* (Phosphofurin Acidic Cluster Sorting Protein 1)	Protein Coding	Schuurs–Hoeijmakers Syndrome and Orthostatic Intolerance
*FHIT* (Fragile Histidine Triad Diadenosine Triphosphatase)	Protein Coding	Renal Cell Carcinoma, Nonpapillary and Sporadic Breast Cancer
*MX1* (MX Dynamin Like GTPase 1)	Protein Coding	Influenza and Subacute Sclerosing Panencephalitis (SSPE)
LINC00456	Long Intergenic Non-Protein Coding RNA 456	
*CHSY1* (Chondroitin Sulfate Synthase 1)	Protein Coding	Temtamy Preaxial Brachydactyly Syndrome and Brachydactyly
*LZTS2* (Leucine Zipper Tumor Suppressor 2)	Protein Coding	

**Table 5:** Performance of individual classifiers and combined max-competing classifiers using blood sample dataset GSE219037 to classify asymptomatic Marines and healthy controls into their respective groups. CF1 and CF2 are two different classifiers. CFmax = max(CF1–2) is the combined max-competing classifier. The numbers are fitted coefficient values.

Sites	Gene	CF1	CF2	CFmax
	Intercept	50.86	−78.93	
cg16279999	*TESC*		110.32	
cg24002522	*ALCAM*	−42.02		
cg00324510	*PACS1*			
cg08949406	*FHIT*	52.38		
cg16785077	*MX1*	−77.76		
cg23933458	*LINC00456*	−0.20	23.19	
cg24002003	*CHSY1*		−67.43	
cg24760467	*LZTS2*		−36.30	
Accuracy	%	80.61	80.23	92.44
Sensitivity	%	61.33	56.0	88.0
Specificity	%	95.88	98.97	95.88

**Table 6:** Performance of individual classifiers and combined max-competing classifiers using blood sample dataset GSE193879 to classify MIS-C patients and COVID-19+ patients into their respective groups. CF1 and CF2 are two different classifiers. CFmax = max(CF1-2) is the combined max-competing classifier. The numbers are fitted coefficient values.

Sites	Gene	CF1	CF2	CFmax
	Intercept	−27.9687	−22.7434	
cg16279999	*TESC*			
cg24002522	*ALCAM*			
cg00324510	*PACS1*	24.7071		
cg08949406	*FHIT*			
cg16785077	*MX1*	18.5328	25.1451	
cg23933458	*LINC00456*		1.9181	
cg24002003	*CHSY1*	−8.986	23.7035	
cg24760467	*LZTS2*			
Accuracy	%	89.66	75.86	94.83
Sensitivity	%	88.37	69.77	95.35
Specificity	%	93.33	93.33	93.33

**Table 7:** Performance of individual classifiers and combined max-competing classifiers using blood sample dataset GSE179325 to classify COVID-19+ patients and healthy controls into their respective groups. CF1-3 are three different classifiers. CFmax = max(CF1-3) is the combined max-competing classifier. The numbers are fitted coefficient values.

Sites	Gene	CF1	CF2	CF3	CFmax
	Intercept	−30.937	−12.3248	−22.4222	
cg16279999	*TESC*		40.5278	35.5007	
cg24002522	*ALCAM*		−3.6707		
cg00324510	*PACS1*	6.4129			
cg08949406	*FHIT*	29.0715			
cg16785077	*MX1*		−31.7679		
cg23933458	*LINC00456*	6.3832			
cg24002003	*CHSY1*			−30.5158	
cg24760467	*LZTS2*			−14.5514	
Accuracy	%	28.57	71.60	48.26	79.79
Sensitivity	%	13.32	68.50	38.48	78.86
Specificity	%	100.00	86.14	94.06	84.16

**Table 8:** Performance of individual classifiers and combined max-competing classifiers using blood sample dataset GSE179325 to classify severe COVID-19+ patients and healthy controls into their respective groups. CF1-3 are three different classifiers. CFmax = max(CF1-3) is the combined max-competing classifier. The numbers are fitted coefficient values.

Sites	Gene	CF1	CF2	CF3	CFmax
	Intercept	−3.0854	−58.5936	−19.8489	
cg16279999	*TESC*		23.3306		
cg24002522	*ALCAM*				
cg00324510	*PACS1*	23.3655		31.8937	
cg08949406	*FHIT*		54.0804		
cg16785077	*MX1*	−25.4411		−8.7879	
cg23933458	*LINC00456*			−8.6934	
cg24002003	*CHSY1*		−24.5055		
cg24760467	*LZTS2*	−1.0033			
Accuracy	%	69.16	85.51	78.97	90.19
Sensitivity	%	45.13	83.19	67.26	94.69
Specificity	%	96.04	88.12	92.08	85.15

**Table 9:** Performance of individual classifiers and combined max-competing classifiers using blood sample dataset GSE179325 to classify mild COVID-19+ patients and healthy controls into their respective groups. CF1-3 are three different classifiers. CFmax = max(CF1-3) is the combined max-competing classifier. The numbers are fitted coefficient values.

Sites	Gene	CF1	CF2	CF3	CFmax
	Intercept	−8.6838	−1.672	−20.4306	
cg16279999	*TESC*	22.8237			
cg24002522	*ALCAM*				
cg00324510	*PACS1*	5.7074	9.0597	11.1895	
cg08949406	*FHIT*			10.1356	
cg16785077	*MX1*	−23.5915			
cg23933458	*LINC00456*				
cg24002003	*CHSY1*		−17.2585		
cg24760467	*LZTS2*		−9.3648	6.3264	
Accuracy	%	74.40	34.49	27.11	80.26
Sensitivity	%	72.22	17.22	8.33	80.83
Specificity	%	82.18	96.04	94.06	78.22

**Table 10:** Performance of individual classifiers and combined max-competing classifiers using blood sample dataset GSE179325 to classify severe and mild COVID-19+ patients into their respective groups. CF1-3 are three different classifiers. CFmax = max(CF1-3) is the combined max-competing classifier. The numbers are fitted coefficient values.

Sites	Gene	CF1	CF2	CF3	CFmax
	Intercept	20.3765	46.7137	−11.3025	
cg16279999	*TESC*	−23.7059			
cg24002522	*ALCAM*			6.9659	
cg00324510	*PACS1*		−37.5048		
cg08949406	*FHIT*		−21.3486		
cg16785077	*MX1*	−1.9268		−2.1241	
cg23933458	*LINC00456*			31.2084	
cg24002003	*CHSY1*	11.1234	11.3969		
cg24760467	*LZTS2*				
Accuracy	%	34.25	66.38	64.48	77.80
Sensitivity	%	14.72	59.72	58.33	77.22
Specificity	%	96.46	87.61	84.07	79.65

**Table 11:** Performance of individual classifiers and combined max-competing classifiers using blood sample dataset GSE174818 to classify hospitalized COVID-19 patients and other types of patients (as control) into their respective groups. CF1-3 are three different classifiers. CFmax = max(CF1-3) is the combined max-competing classifier. The numbers in (**a**–**e**) are fitted coefficient values. (**f**) presents coefficient signs in (**a**–**e**). Negative signs are expressed as ‘n’, and positive signs are indicated as ‘p’. (**g**) combines (**a**–**e**) to form a new combination with each individual classifier having the same and highest accuracy.

(a)																
Sites		Gene			CF1			CF2			CF3			CFmax		
		Intercept			−107.538			75.7212			109.6635					
cg03870777		*KRT8*			−63.7927											
cg16279999		*TESC*			124.0192											
cg15528722		*TTC33*			−5.9786											
cg24596788		*LOC100422212*						−114.038			−69.9951					
cg11186858		*SEC14L1*						9.1088								
cg24002522		*ALCAM*						42.4985								
cg22930808		*PARP9*														
cg25932713		*PARP9*									−87.3865					
cg26301516		*FNDC3B*									43.7713					
Accuracy					83.59%			72.66%			68.75%			100%		
Sensitivity					79.41%			65.69%			60.78%			100%		
Specificity					100%			100%			100%			100%		
**(b)**																
**Sites**		**Gene**			**CF1**			**CF2**			**CF3**			**CFmax**		
		Intercept			−0.0592			16.3317			−59.7148					
cg03870777		*KRT8*			−73.3841											
cg16279999		*TESC*									40.8761					
cg15528722		*TTC33*			−10.95											
cg24596788		*LOC100422212*						−42.3716								
cg11186858		*SEC14L1*			21.7146											
cg24002522		*ALCAM*														
cg22930808		*PARP9*						−6.2028			−3.5323					
cg25932713		*PARP9*														
cg26301516		*FNDC3B*						35.6417			27.1637					
Accuracy					82.81%			88.28%			50%			100%		
Sensitivity					78.43%			85.29%			37.25%			100%		
Specificity					100%			100%			100%			100%		
**(c)**																
**Sites**		**Gene**			**CF1**			**CF2**			**CF3**			**CFmax**		
		Intercept			57.4536			−100.631			2.9006					
cg03870777		*KRT8*									−83.4044					
cg16279999		*TESC*						79.6075			52.984					
cg15528722		*TTC33*														
cg24596788		*LOC100422212*			−81.6208											
cg11186858		*SEC14L1*														
cg24002522		*ALCAM*						−11.2572								
cg22930808		*PARP9*			−13.8329											
cg25932713		*PARP9*									−51.6332					
cg26301516		*FNDC3B*			34.5399			41.5304								
Accuracy					89.84%			58.59%			67.19%			100%		
Sensitivity					87.25%			48.04%			58.82%			100%		
Specificity					100%			100%			100%			100%		
**(d)**																
**Sites**		**Gene**			**CF1**			**CF2**			**CF3**			**CFmax**		
		Intercept			−104.466			−10.3324			4.7928					
cg03870777		*KRT8*									−87.6922					
cg16279999		*TESC*			88.866											
cg15528722		*TTC33*			−14.4221			−18.1529			−13.0186					
cg24596788		*LOC100422212*														
cg11186858		*SEC14L1*									19.7615					
cg24002522		*ALCAM*						57.7129								
cg22930808		*PARP9*														
cg25932713		*PARP9*						−22.7502								
cg26301516		*FNDC3B*			38.9308											
Accuracy					89.84%			60.94%			89.84%			100%		
Sensitivity					87.25%			50.98%			87.25%			100%		
Specificity					100%			100%			100%			100%		
**(e)**																
**Sites**		**Gene**			**CF1**			**CF2**			**CF3**			**CFmax**		
		Intercept			−47.6492			60.9853			−63.9485					
cg03870777		*KRT8*									−61.9501					
cg16279999		*TESC*						85.7758			65.8907					
cg15528722		*TTC33*			−8.1331			−16.9343								
cg24596788		*LOC100422212*						−138.010								
cg11186858		*SEC14L1*														
cg24002522		*ALCAM*			11.7749						11.7696					
cg22930808		*PARP9*														
cg25932713		*PARP9*														
cg26301516		*FNDC3B*			58.4412											
Accuracy					89.84%			60.94%			89.84%			100%		
Sensitivity					87.25%			50.98%			87.25%			100%		
Specificity					100%			100%			100%			100%		
**(f)**																
**Sites**	**Gene**	**(a) CF-**			**(b) CF-**			**(c) CF-**			**(d) CF-**			**(e) CF-**		
		1	2	3	1	2	3	1	2	3	1	2	3	1	2	3
cg03870777	*KRT8*	n			n					n			n			n
cg16279999	*TESC*	p					p		p	p	p				p	p
cg15528722	*TTC33*	n			n						n	n	n	n	n	
cg24596788	*LOC100422212*		n	n		n		n							n	
cg11186858	*SEC14L1*		p		p								p			p
cg24002522	*ALCAM*		p						n			p		p		
cg22930808	*PARP9*					n	n	n								
cg25932713	*PARP9*			n						n		n				
cg26301516	*FNDC3B*			p		p	p	p	p		p			p		
**(g)**																
**Sites**		**Gene**			**CF1**			**CF2**			**CF3**			**CFmax**		
		Intercept			−104.466			57.4536			4.7928					
cg03870777		*KRT8*									−87.6922					
cg16279999		*TESC*			88.866											
cg15528722		*TTC33*			−14.4221						−13.0186					
cg24596788		*LOC100422212*						−81.6208								
cg11186858		*SEC14L1*									19.7615					
cg24002522		*ALCAM*														
cg22930808		*PARP9*						−13.8329								
cg25932713		*PARP9*														
cg26301516		*FNDC3B*			38.9308			34.5399								
Accuracy					89.84%			89.84%			89.84%			100%		
Sensitivity					87.25%			87.25%			87.25%			100%		
Specificity					100%			100%			100%			100%		

**Table 12:** Descriptions of genes in Table 11.

Gene Name	Gene Type	Associated Diseases
KRT8 (Keratin 8)	Protein Coding	Cyrhosis and Hidrocystoma
*TTC33* (Tetratricopeptide Repeat Domain 33)	Protein Coding	Mixed Fibrolamellar Hepatocellular Carcinoma
*LOC100422212* (Eukaryotic Translation Initiation Factor 3 Subunit J Pseudogene)	Pseudogene	
*SEC14L1* (SEC14 Like Lipid Binding 1)	Protein Coding	Complement Component 7 Deficiency and Phlyctenulosis
*PARP9* (Poly(ADP-Ribose) Polymerase Family Member 9)	Protein Coding	Lymphoma and B-Cell Lymphoma
*FNDC3B* (Fibronectin Type III Domain Containing 3B)	Protein Coding	Urethra Clear Cell Adenocarcinoma and Urethra Adenocarcinoma

**Table 13:** TQCC-based beta values: Performance of individual classifiers and combined max-competing classifiers using blood sample dataset GSE174818 to classify hospitalized COVID-19 patients and other types of patients (as control) into their respective groups. CF1-3 are three different classifiers. CFmax = max(CF1-3) is the combined max-competing classifier. The numbers are fitted coefficient values.

Sites	Gene	CF1	CF2	CF3	CF4	CF5	CFmax
	Intercept	−32.2284	6.8728	−33.4684	13.8944	−15.4975	
cg02434330	*PYCARD*				−46.0883		
cg03926906					−58.3508		
cg20608348	*SPON2*			−127.171			
cg25634004	*DENND1B*			28.2635		39.2045	
cg03044471	*TOLLIP*		−62.2568				
cg22894824	*PAM*	−130.721	−169.084				
cg08028503	*RP11-326C3.11*	36.021					
cg12252979			−10.929				
cg18679416						−15.8555	
cg03587597	*LY6E*	55.1923					
cg24265195	*DYSF*			28.0894			
cg13753515	*SIGLEC1*					−78.4003	
cg07133321	*LIMD1*				−39.6183		
Accuracy	%	69.53	24.22	67.19	53.91	71.88	100.00
Sensitivity	%	61.76	4.90	58.82	42.16	64.71	100.00
Specificity	%	100.00	100.00	100.00	100.00	100.00	100.00

**Table 14:** Descriptions of genes in Table 13.

Gene Name	Gene Type	Associated Diseases
*PYCARD* (PYD And CARD Domain Containing)	Protein Coding	Familial Mediterranean Fever and Osteomyelitis
*SPON2* (Spondin 2)	Protein Coding	Pharyngitis
*DENND1B* (DENN Domain Containing 1B)	Protein Coding	Asthma and Childhood-Onset Asthma
*TOLLIP* (Toll Interacting Protein)	Protein Coding	Tick Paralysis and Acute Vascular Insufficiency of Intestine
*PAM* (Peptidylglycine Alpha-Amidating Monooxygenase)	Protein Coding	Menkes Disease and Spinal Muscular Atrophy
*LY6E* (Lymphocyte Antigen 6 Family Member E)	Protein Coding	Acute Promyelocytic Leukemia
*DYSF* (Dysferlin)	Protein Coding	Miyoshi Muscular Dystrophy 1 and Muscular Dystrophy, Limb-Girdle, Autosomal Recessive 2
*SIGLEC1* (Sialic Acid Binding Ig Like Lectin 1)	Protein Coding	Rheumatoid Arthritis and Arthritis
*LIMD1* (LIM Domain Containing 1)	Protein Coding	Lung Cancer and Breast Cancer

**Table 15:** Performance of individual classifiers and combined max-competing classifiers using blood sample dataset GSE172114 to classify critical COVID-19 young patients and non-patients (as control) into their respective groups. CF1-4 are four different classifiers. CFmax = max(CF1–2) or CFmax = max(CF3–4) is the combined max-competing classifier. The numbers are fitted coefficient values.

	CF1–CF2 Combination			CF3–CF4 Combination		
Gene	CF1	CF2	CFmax	CF3	CF4	CFmax
Intercept	−30.4307	−19.207		−12.7685	−9.7796	
*GCKR*		4.0816		−0.1638	8.401	
*HNRNPLL*	3.9282	4.1291			3.5237	
*METTL9*	0.0892			1.6781		
Accuracy%	86.96	85.51	98.55	86.96	84.06	98.55
Sensitivity%	82.61	78.26	100.00	82.61	76.09	100.00
Specificity%	95.65	100.00	95.65	95.65	100.00	95.65

## Data Availability

The datasets are publicly available. The data links are stated in Section Data Description. Computing outputs are in a supplementary file available online https://pages.stat.wisc.edu/~zjz/DNAmethylation02.zip (accessed on 22 November 2022) or available making email requests to the authors. The results presented in this paper are all verifiable by simply checking the Excel sheets and formulas in the file.
